# Risk of diabetic retinopathy and diabetic macular oedema with sodium–glucose cotransporter 2 inhibitors and glucagon-like peptide 1 receptor agonists in type 2 diabetes: a real-world data study from a global federated database

**DOI:** 10.1007/s00125-024-06132-5

**Published:** 2024-04-08

**Authors:** Aikaterini Eleftheriadou, David Riley, Sizheng S. Zhao, Philip Austin, Gema Hernández, Gregory Y. H. Lip, Timothy L. Jackson, John P. H. Wilding, Uazman Alam

**Affiliations:** 1 Department of Cardiovascular & Metabolic Medicine, Institute of Life Course and Medical Sciences, University of Liverpool, Liverpool, UK; 2 Centre for Musculoskeletal Research, Division of Musculoskeletal and Dermatological Science, School of Biological Sciences, Faculty of Biological Medicine and Health, University of Manchester, Manchester Academic Health Science Centre, Manchester, UK; 3 TriNetX LLC, Cambridge, MA USA; 4 Liverpool Centre for Cardiovascular Science at the University of Liverpool, Liverpool John Moores University and Liverpool Heart & Chest Hospital, Liverpool, UK; 5 Danish Center for Health Services Research, Department of Clinical Medicine, Aalborg University, Aalborg, Denmark; 6 Faculty of Life Science and Medicine, King’s College London, London, UK; 7 King’s Ophthalmology Research Unit (KORU), King’s College Hospital, London, UK; 8 Department of Medicine, University Hospital Aintree, Liverpool University NHS Foundation Trust, Liverpool, UK; 9 Visiting Fellow, Centre for Biomechanics and Rehabilitation Technologies, Staffordshire University, Stoke-on-Trent, UK

**Keywords:** Clinical diabetes, Microvascular disease, Retinopathy

## Abstract

**Aims/hypothesis:**

A protective role of sodium–glucose cotransporter 2 inhibitors (SGLT2is) and glucagon-like peptide 1 receptor agonists (GLP1-ra) in the development of diabetic retinopathy and diabetic macular oedema has been described in some recent studies, which may extend beyond glycaemic control. We aimed to review the clinical impact of SGLT2i and GLP1-ra therapy on the risk of diabetic retinopathy and diabetic macular oedema in individuals with type 2 diabetes taking insulin.

**Methods:**

This is a retrospective cohort analysis of approximately two million people with type 2 diabetes receiving insulin across 97 healthcare organisations using a global federated health research network (TriNetX, Cambridge, USA). Two intervention cohorts (SGLT2i + insulin, *n*=176,409; GLP1-ra + insulin, *n*=207,034) were compared against a control cohort (insulin with no SGLT2i/GLP1-ra, *n*=1,922,312). Kaplan–Meier survival analysis was performed and estimated HRs were reported for each outcome. Propensity score was used to 1:1 match for age, sex, ischaemic heart disease, hypertension, microvascular complications, chronic kidney disease, HbA_1c_, BMI and use of pioglitazone, lipid modifying agents, antilipemic agents, ACE inhibitors, angiotensin II inhibitors and metformin. A sub-analysis comparing the two intervention cohorts was also performed.

**Results:**

SGLT2i with insulin was associated with a reduced HR (95% CI) for diabetic macular oedema compared with the control cohort (0.835; 0.780, 0.893), while GLP1-ra with insulin demonstrated a lack of signal with no statistical significance to the HR (1.013; 0.960, 1.069). SGLT2i with insulin was not associated with a clinically significant increase in the risk of developing diabetic retinopathy (1.076; 1.027, 1.127), while GLP1-ra with insulin increased diabetic retinopathy risk (1.308; 1.261, 1.357). Compared with SGLT2i with insulin, GLP1-ra with insulin was associated with higher risk of diabetic retinopathy (1.205; 1.153, 1.259) and diabetic macular oedema (1.130; 1.056, 1.208).

**Conclusions/interpretation:**

Our study suggests that the combination of SGLT2i and insulin is associated with lower risk of developing diabetic macular oedema. However, the use of GLP1-ra was associated with an increased risk of diabetic retinopathy in individuals with type 2 diabetes also taking insulin. A comparative analysis showed favourable outcomes with SGLT2i and insulin in the development of diabetic macular oedema and diabetic retinopathy. RCTs using dedicated  retinal imaging are required to determine the causal relationship with these therapies.

**Graphical Abstract:**

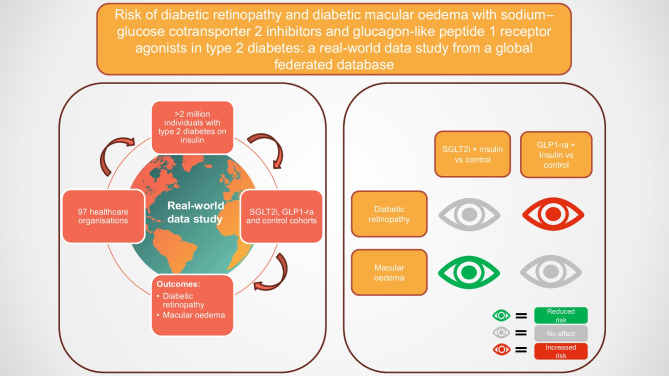

**Supplementary Information:**

The online version contains peer-reviewed but unedited supplementary material available at 10.1007/s00125-024-06132-5.


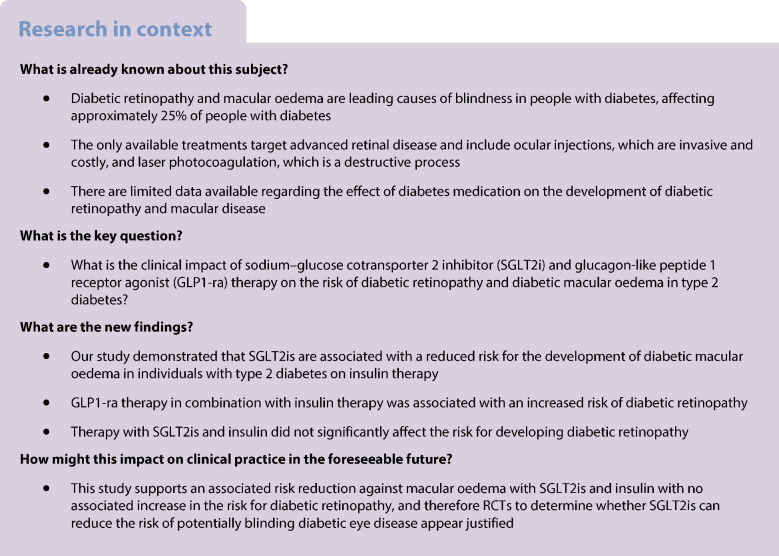


## Introduction

Diabetes mellitus affects 463 million people worldwide and this number is projected to rise to 700 million by 2045, making it a major global epidemic [1]. In the UK, 4.9 million individuals have diabetes, with £10 billion annually allocated by the National Health Service [2, 3]. Diabetic retinopathy and diabetic macular oedema (DMO) are leading causes of vision loss in the diabetic population [4]. The Wisconsin Epidemiologic Study of Diabetic Retinopathy XV notes a concerning progression in two-thirds of individuals who had diabetic retinopathy at baseline to more severe stages of diabetic retinopathy, and 20% to proliferative diabetic retinopathy and DMO [5]. While treatments for proliferative diabetic retinopathy and vision-threatening DMO such as intravitreal anti-vascular endothelial growth factor (VEGF) injections exist, their invasiveness, cost and complications pose challenges [6]. Despite the current focus on glycaemic control, the multifactorial nature of diabetic retinopathy and DMO suggests unidentified risk factors and potential new therapeutic targets [7].

Glucagon-like peptide 1 receptor agonists (GLP1-ra) have been used for over a decade in the management of type 2 diabetes [8]. Other than improving glycaemic control, GLP1-ra have positive effects on body weight, and a number of GLP1-ra agents have shown cardiovascular (CV), reno-protective and mortality benefits. Similarly, sodium–glucose cotransporter 2 inhibitors (SGLT2is) are a more recent class of medication now firmly established in diabetes management. Similar to GLP1-ra, the therapeutic effect of SGLT2is has been re-appraised with dedicated CV and renal outcome trials, with notable benefits even in the absence of type 2 diabetes [9]. Additionally, recent studies have suggested a possible role for both GLP1-ra and SGLT2is in the management of diabetic retinopathy and DMO that extends beyond glycaemic control [10, 11]. Possible mechanisms include the reduction of retinal endothelial cell apoptosis and mitigation of gradual central retinal thinning with SGLT2i, a neuroprotective role in retinal ganglion cells with GLP1-ra, as well as a reduction in the oxidative stress and vascular remodelling with both medications [12–16]. While CV and renal benefits are widely acknowledged, their potential impact on diabetic retinopathy and DMO, particularly with SGLT2i, is promising but not as firmly established.

In this this real-world study using a global federated dataset, we aimed to evaluate the impact of SGLT2i and GLP1-ra therapy on the risk of diabetic retinopathy and DMO in individuals with type 2 diabetes taking insulin. We compared the impact of SGLT2i vs a control cohort, GLP1-ra vs control cohort and SGLT2i vs GLP1-ra.

## Methods

### Data source

This retrospective cohort study used data acquired from TriNetX (Cambridge, USA), a global federated health research network that has access to electronic medical records (EMRs) from healthcare organisations (HCOs) worldwide. Analyses were conducted on this Global Collaborative Network, which encompasses data from 111 HCOs and over 114 million individuals. This federated data network comprises procedures, medications, laboratory values, genomics and other clinically relevant information generated in EMRs, cancer registries and other departmental systems that HCOs can make available to TriNetX for its harmonisation and analysis [17]. Although HCOs are responsible for the integrity of their data, the data ingestion process includes several data quality checks of cleanliness, consistency, correctness and completeness to capture potential errors in the data. HCOs and TriNetX are continuously monitoring these errors and work together to fix them once identified [18]. All data collection, processing and transmission are performed in compliance with all data protection laws applicable to the contributing HCOs, including the European Union Data Protection Law Regulation 2016/679, the General Data Protection Regulation on the protection of natural persons regarding the processing of personal data, and the Health Insurance Portability and Accountability Act, the US federal law that protects the privacy and security of healthcare data. As detailed in our previous publication, the TriNetX Global Collaborative Network is a distributed network (with most HCOs located in the USA), and analytics are performed at the HCO with only aggregate results being surfaced and returned to the platform [19]. All HCOs are in agreement with data usage and publication laws.

### Study population

For this study, ~2 million people with type 2 diabetes on insulin from 97 HCOs were identified as per the ICD, Tenth Revision, Clinical Modification (ICD-10-CM; https://icd.who.int/browse10/2019/en) code E11 in their EMR. The population used in this study was sourced from TriNetX. TriNetX provides access to EMRs from HCOs worldwide, encompassing essential demographic information such as patients’ age, sex/gender, and regional origins (including data from the US, EMEA (Europe, Middle East and Africa), LATAM (Latin America) and APAC (Asia Pacific) regions). Socioeconomic and ethnicity factors are often regarded as highly sensitive data and consequently are not typically included in the information provided by many HCOs. Inclusion criteria comprised individuals over the age of 18, with a diagnosis of type 2 diabetes (ICD-10-CM E11) and on insulin therapy (as duration/severity of disease cannot be identified through TriNetX and to reduce the extent of confounding by indication). Type 1 diabetes (ICD-10-CM code E10) and diabetic neuropathy (ICD-10-CM codes E11.40, E11.41 and E11.42) were used as exclusion criteria when building the cohorts, the latter to balance for diabetic complications amongst the cohorts. Three cohorts were identified from the results obtained: (1) control (insulin and no SGLT2i/GLP1-ra); (2) SGLT2i (and insulin, not on GLP1-ra); and (3) GLP1-ra (and insulin, not on SGLT2i). The three analyses conducted were: (1) SGLT2i vs control; (2) GLP1-ra vs control; and (3) SGLT2i vs GLP1-ra. Each analysis was propensity score matched for age; sex; use of pioglitazone, lipid modifying agents, antilipemic agents, ACE inhibitors, angiotensin II inhibitors and metformin; presence of ischaemic heart disease (ICD-CM-10, I20–25) and essential hypertension (I10); presence of microvascular complications (nephropathy [E11.2], retinopathy [E11.31–35] and neuropathy [E11.40–43]); CKD stage (through relevant eGFR value groups); BMI (≤30 kg/m^2^ and >30 kg/m^2^); and HbA_1c_ value (≤53 mmol/mol and >53 mmol/mol [≤7% and >7%]). The propensity score matching algorithm used 1:1 matching. Participants were matched with a ‘greedy nearest neighbour matching’ algorithm with a calliper of 0.1. It was not possible to propensity score for HbA_1c_ and BMI as continuous variables; instead, participants were matched into two HbA_1c_ and BMI categories as mentioned above. The HbA_1c_, BMI and eGFR values were based on the most recent values for each participant when the treatment started.

### Outcome analysis

The start date of the analysis was 1 January 2010 and the end date was 21 December 2023, defining the time window. Individuals who had an index date within the above time window were eligible to be included in the analysis. The index date was defined as the initiation of insulin in the control cohort, the co-prescription of insulin and SGLT2i (in any order) in the SGLT2i cohort and the co-prescription of insulin and GLP1-ra (in any order) in the GLP1-ra cohort. The length of treatment could not be identified through TriNetX, and thus the study effectively conducted an intention-to-treat analysis. The outcomes were determined by the incidence of the relevant condition, identified as the first occurrence of the corresponding clinical ICD-10 coding within 5 years from the index date or by the end date of the study, in the respective cohorts.

The main outcomes for this study were: development of diabetic retinopathy (ICD-10 codes: E11.319, E11.329, E11.339, E11.349, E11.359, E11.352, E11.353, E11.354, E11.355) and DMO (ICD-10 codes: H35.81, E11.311, E11.321, E11.331, E11.341, E11.351). Positive control outcomes of heart failure (HF) (ICD-10 code: I50), hospitalisation (from any aetiology) and all-cause mortality (ICD-10 code: R99) were included in the analysis to test that our observational approach can provide estimates that match causal effects that are already well documented and demonstrated in randomised clinical trials. Individuals with a specific outcome prior to the index date were excluded from the specific analysis for this outcome (e.g. individuals with retinopathy prior to the index event were excluded from the retinopathy outcome analysis, etc.).

### Statistical analysis

Using the TriNetX software (a bespoke proprietary solution tailored for application within the clinical research sphere; TriNetX, USA), Kaplan–Meier survival analysis was performed. The Kaplan–Meier analysis estimates probability of the outcome at a respective time interval (daily time interval was used in this analysis). In order to account for the individuals who exited the cohort during the analysis period, and therefore should not be included in the analysis, censoring was applied. In this analysis, participants were removed from the analysis (censored) after the last entry in their record. The data included the following: the number of participants in each cohort; the number of participants with the aforementioned outcomes; median survival; and survival probability at the end of the time window. In the TriNetX platform, the HRs and their associated CIs and *χ*^2^ values were calculated in each analysis using R’s Survival package [20] and were validated by comparing them with the output from SAS. The Cox proportional hazards model was used in which the only covariate for a participant was their cohort membership. Survival curves were observed for crossing over between the comparison cohorts. A large *χ*^2^ value is associated with low proportionality and a small *χ*^2^ value with high proportionality. Boundary numbers are not robust across different industry-standard software packages so the interpretation is qualitative.

E-value calculations were performed to establish the minimum strength of association, on the HR, that an unmeasured confounder would need to have with both the exposure and the outcome, conditional on the measured covariates, to fully explain away a specific exposure–outcome association, as described by VanderWeele and Ding [21]. A high E-value indicates that substantial unmeasured confounding would be necessary to dismiss an effect estimate, while a low E-value suggests minimal unmeasured confounding is needed for the same purpose.

## Results

### Study population

**Fig. 1:**
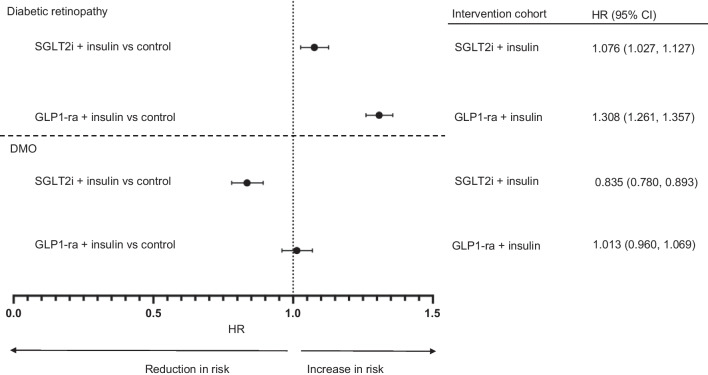
HRs for diabetic retinopathy and DMO for the SGLT2i + insulin and GLP1-ra + insulin cohorts vs control cohorts

From 1 January 2010 to 21 December 2023, 176,409 individuals with type 2 diabetes receiving SGLT2i plus insulin, 207,034 receiving GLP1-ra plus insulin and 1,922,312 individuals receiving insulin but no SGLT1i/GLP1-ra were identified. After propensity score matching for the aforementioned characteristics and confounders, the baseline characteristics were well balanced amongst all cohorts (Tables 1, 2 and 3, electronic supplementary material [ESM] Fig. 1).

**Table 1:** Propensity score matching (PSM) for the SGLT2i + insulin vs control cohorts analysis

	Before PSM		After PSM	
	SGLT2i + insulin	Control	SGLT2i + insulin	Control
Total number of participants	174,484	1,895,554	154,538	154,538
Demographics				
Age at index	64.1±12.4	64.0±14.5	63.9±12.5	64.1±12.8
Female	68,120 (39.0)	894,244 (47.2)	60,343 (39.0)	60,227 (39.0)
Male	102,253 (58.6)	974,171 (51.4)	90,484 (58.6)	90,716 (58.7)
BMI≤30 kg/m^2^	33,321 (19.1)	225,662 (11.9)	28,159 (18.2)	27,169 (17.6)
BMI>30 kg/m^2^	39,758 (22.8)	253,637 (13.4)	33,431 (21.6)	33,020 (21.4)
Diagnosis				
Ischaemic heart diseases	71,866 (41.2)	369,872 (19.5)	60,338 (39.0)	60,437 (39.1)
Kidney complications	37,989 (21.8)	143,147 (7.6)	29,314 (19.0)	28,496 (18.4)
Unspecified diabetic retinopathy	6437 (3.7)	24,909 (1.3)	4245 (2.7)	3875 (2.5)
Mild nonproliferative diabetic retinopathy	4540 (2.6)	11,805 (0.6)	3160 (2.0)	2847 (1.8)
Moderate nonproliferative diabetic retinopathy	1668 (1.0)	3646 (0.2)	1046 (0.7)	895 (0.6)
Severe nonproliferative diabetic retinopathy	682 (0.4)	1672 (0.1)	412 (0.3)	369 (0.2)
Proliferative diabetic retinopathy	2237 (1.3)	8817 (0.5)	1526 (1.0)	1426 (0.9)
Diabetic neuropathy, unspecified	12,438 (7.1)	0	0	0
Diabetic mononeuropathy	444 (0.3)	0	91 (0.1)	0
Diabetic polyneuropathy	11,083 (6.4)	0	10 (0)	0
Diabetic autonomic (poly)neuropathy	2071 (1.2)	4848 (0.3)	1077 (0.7)	865 (0.6)
Essential hypertension	125,116 (71.7)	858,232 (45.3)	107,373 (69.5)	97,361 (63.0)
Medication				
Pioglitazone	11,663 (6.7)	35,593 (1.9)	9769 (6.3)	10,729 (6.9)
Lipid modifying agents	127,358 (73.0)	541,987 (28.6)	109,937 (71.1)	72,219 (46.7)
Antilipemic agents	127,548 (73.1)	541,606 (28.6)	110,114 (71.3)	72,210 (46.7)
ACE inhibitors	76,511 (43.8)	335,634 (17.7)	64,898 (42.0)	47,811 (30.9)
Angiotensin II inhibitors	62,705 (35.9)	203,240 (10.7)	54,141 (35.0)	26,952 (17.4)
Metformin	99,478 (57.0)	363,083 (19.2)	86,018 (55.7)	51,427 (33.3)
Laboratory				
HbA_1c_≤53 mmol/mol^**a**^	74,393 (42.6)	451,360 (23.8)	63,818 (41.3)	64,912 (42.0)
HbA_1c_>53 mmol/mol^**a**^	97,575 (55.9)	330,006 (17.4)	82,300 (53.3)	83,364 (53.9)
eGFR>90 ml/min per 1.73m^2^	81,495 (46.7)	492,441 (26.0)	70,374 (45.5)	69,586 (45.0)
eGFR 60–90 ml/min per 1.73m^2^	110,890 (63.6)	683,710 (36.1)	95,289 (61.7)	95,187 (61.6)
eGFR 45–60 ml/min per 1.73m^2^	72,015 (41.3)	400,018 (21.1)	60,044 (38.9)	59,963 (38.8)
eGFR 30–45 ml/min per 1.73m^2^	45,862 (26.3)	250,958 (13.2)	37,265 (24.1)	37,097 (24.0)
eGFR 0–30 ml/min per 1.73m^2^	28,351 (16.2)	201,887 (10.7)	22,885 (14.8)	21,731 (14.1)

Data are *n* (%) or mean±SD

^a^HbA_1c_ 53 mmol/mol is equivalent to 7%

**Table 2:** Propensity score matching (PSM) for the GLP1-ra + insulin vs control cohorts analysis

	Before PSM		After PSM	
	GLP1-ra + insulin	Control	GLP1-ra + insulin	Control
Total number of participants	206,387	1,895,554	183,091	183,091
Demographics				
Age at index	58.6±13.2	64.0±14.5	58.3±13.4	58.3±13.9
Female	115,053 (55.7)	894,244 (47.2)	102,349 (55.9)	102,928 (56.2)
Male	86,748 (42.0)	974,171 (51.4)	76,433 (41.7)	75,969 (41.5)
BMI≤30 kg/m^2^	24,563 (11.9)	225,662 (11.9)	20,872 (11.4)	19,411 (10.6)
BMI>30 kg/m^2^	53,222 (25.8)	253,637 (13.4)	45,792 (25.0)	44,936 (24.5)
Diagnosis				
Ischaemic heart diseases	45,127 (21.9)	369,872 (19.5)	36,441 (19.9)	35,984 (19.7)
Kidney complications	32,888 (15.9)	143,147 (7.6)	24,221 (13.2)	23,159 (12.6)
Unspecified diabetic retinopathy	8714 (4.2)	24,909 (1.3)	5687 (3.1)	5276 (2.9)
Mild nonproliferative diabetic retinopathy	6207 (3.0)	11,805 (0.6)	4142 (2.3)	3751 (2.0)
Moderate nonproliferative diabetic retinopathy	2176 (1.1)	3646 (0.2)	1312 (0.7)	1179 (0.6)
Severe nonproliferative diabetic retinopathy	829 (0.4)	1672 (0.1)	494 (0.3)	414 (0.2)
Proliferative diabetic retinopathy	2734 (1.3)	8817 (0.5)	1766 (1.0)	1558 (0.9)
Diabetic neuropathy, unspecified	14,405 (7.0)	0	0	0
Diabetic mononeuropathy	588 (0.3)	0	27 (0.0)	0
Diabetic polyneuropathy	13,438 (6.5)	0	0	0
Diabetic autonomic (poly)neuropathy	2245 (1.1)	4848 (0.3)	1143 (0.6)	864 (0.5)
Essential hypertension	138,757 (67.2)	858,232 (45.3)	118,230 (64.6)	100,827 (55.1)
Medication				
Pioglitazone	14,235 (6.9)	35,593 (1.9)	12,020 (6.6)	12,821 (7.0)
Lipid modifying agents	132,519 (64.2)	541,987 (28.6)	113,370 (61.9)	70,208 (38.3)
Antilipemic agents	132,781 (64.3)	541,606 (28.6)	113,611 (62.1)	70,104 (38.3)
ACE inhibitors	83,971 (40.7)	335,634 (17.7)	71,030 (38.8)	48,936 (26.7)
Angiotensin II inhibitors	55,393 (26.8)	203,240 (10.7)	47,464 (25.9)	26,118 (14.3)
Metformin	124,688 (60.4)	363,083 (19.2)	108,643 (59.3)	62,840 (34.3)
Laboratory				
HbA_1c_≤53 mmol/mol^**a**^	86,923 (42.1)	451,360 (23.8)	75,189 (41.1)	75,648 (41.3)
HbA_1c_>53 mmol/mol^**a**^	118,403 (57.4)	330,006 (17.4)	100,269 (54.8)	100,795 (55.1)
eGFR>90 ml/min per 1.73m^2^	96,567 (46.8)	492,441 (26.0)	83,859 (45.8)	82,828 (45.2)
eGFR 60–90 ml/min per 1.73m^2^	116,525 (56.5)	683,710 (36.1)	99,712 (54.5)	99,214 (54.2)
eGFR 45–60 ml/min per 1.73m^2^	64,507 (31.3)	400,018 (21.1)	52,898 (28.9)	52,386 (28.6)
eGFR 30–45 ml/min per 1.73m^2^	38,174 (18.5)	250,958 (13.2)	30,350 (16.6)	29,690 (16.2)
eGFR 0–30 ml/min per 1.73m^2^	26,219 (12.7)	201,887 (10.7)	20,700 (11.3)	19,444 (10.6)

Data are *n* (%) or mean±SD

^a^HbA_1c_ 53 mmol/mol is equivalent to 7%

**Table 3:** Propensity score matching (PSM) for the GLP1-ra + insulin vs SGLT2i + insulin cohorts analysis

	Before PSM		After PSM	
	GLP1-ra	SGLT2i	GLP1-ra	SGLT2i
Total number of participants	206,387	174,484	139,117	139,117
Demographics				
Age at index	58.6±13.2	64.1±12.4	62.1±11.9	62.1±12.3
Female	115,053 (55.7)	68,120 (39.0)	61,948 (44.5)	62,776 (45.1)
Male	86,748 (42.0)	102,253 (58.6)	73,946 (53.2)	73,099 (52.5)
BMI≤30 kg/m^2^	24,563 (11.9)	33,321 (19.1)	20,472 (14.7)	20,640 (14.8)
BMI>30 kg/m^2^	53,222 (25.8)	39,758 (22.8)	32,907 (23.7)	32,522 (23.4)
Diagnosis				
Ischaemic heart diseases	45,127 (21.9)	71,866 (41.2)	42,219 (30.3)	42,607 (30.6)
Kidney complications	32,888 (15.9)	37,989 (21.8)	25,999 (18.7)	26,221 (18.8)
Unspecified diabetic retinopathy	8714 (4.2)	6437 (3.7)	5361 (3.9)	5564 (4.0)
Mild nonproliferative diabetic retinopathy	6207 (3.0)	4540 (2.6)	3780 (2.7)	3944 (2.8)
Moderate nonproliferative diabetic retinopathy	2176 (1.1)	1668 (1.0)	1363 (1.0)	1449 (1.0)
Severe nonproliferative diabetic retinopathy	829 (0.4)	682 (0.4)	546 (0.4)	593 (0.4)
Proliferative diabetic retinopathy	2734 (1.3)	2237(1.3)	1836 (1.3)	1920 (1.4)
Diabetic neuropathy, unspecified	14,405 (7.0)	12,438 (7.1)	9740 (7.0)	9951 (7.2)
Diabetic mononeuropathy	588 (0.3)	444 (0.3)	351 (0.3)	375 (0.3)
Diabetic polyneuropathy	13,438 (6.5)	11,083 (6.4)	8861 (6.4)	9087 (6.5)
Diabetic autonomic (poly)neuropathy	2245 (1.1)	2071 (1.2)	1616 (1.2)	1600 (1.2)
Essential hypertension	138,757 (67.2)	125,116 (71.7)	97,906 (70.4)	95,733 (68.8)
Medication				
Pioglitazone	14,235 (6.9)	11,663 (6.7)	9800 (7.0)	9673 (7.0)
Lipid modifying agents	132,519 (64.2)	127,358 (73.0)	95,161 (68.4)	97,122 (69.8)
Antilipemic agents	132,781 (64.3)	127,548 (73.1)	95,365 (68.6)	97,286 (69.9)
ACE inhibitors	83,971 (40.7)	76,511 (43.8)	58,983 (42.4)	58,516 (42.1)
Angiotensin II inhibitors	55,393 (26.8)	62,705 (35.9)	40,117 (28.8)	46,639 (33.5)
Metformin	124,688 (60.4)	99,478 (57.0)	82,062 (59.0)	80,354 (57.8)
Laboratory				
HbA_1c_≤53 mmol/mol^**a**^	86,923 (42.1)	74,393 (42.6)	54,734 (39.3)	56,951 (40.9)
HbA_1c_>53 mmol/mol^**a**^	118,403 (57.4)	97,575 (55.9)	78,710 (56.6)	78,807 (56.6)
eGFR>90 ml/min per 1.73m^2^	96,567 (46.8)	81,495 (46.7)	62,392 (44.8)	63,577 (45.7)
eGFR 60–90 ml/min per 1.73m^2^	116,525 (56.5)	110,890 (63.6)	81,530 (58.6)	83,307 (59.9)
eGFR 45–60 ml/min per 1.73m^2^	64,507 (31.3)	72,015 (41.3)	49,994 (35.9)	50,480 (36.3)
eGFR 30–45 ml/min per 1.73m^2^	38,174 (18.5)	45,862 (26.3)	31,009 (22.3)	31,176 (22.4)
eGFR 0–30 ml/min per 1.73m^2^	26,219 (12.7)	28,351 (16.2)	20,024 (14.4)	20,082 (14.4)

Data are *n* (%) or mean±SD

^a^HbA_1c_ 53 mmol/mol is equivalent to 7%

### Main outcome: diabetic retinopathy and macular oedema

The number of participants included in each analysis, number of individuals excluded due to having the outcome prior to the time window, number of events, median survival time and survival probability are displayed in ESM Tables 1–5, which include important information such as sample size and event-related variables for each analysis.

#### SGLT2i + insulin vs control

The HR for diabetic retinopathy was 1.076 (95% CI 1.027, 1.127; *χ*^2^=16.832), which although statistically significant is unlikely to be clinically significant. The HR for DMO was 0.835 (95% CI 0.780, 0.893; *χ*^2^=10.986), indicating a lower risk with SGLT2i and insulin compared with the control cohort (Fig. 1, ESM Figs 2, 3).

#### GLP1-ra + insulin vs control

The HR for diabetic retinopathy was 1.308 (95% CI 1.261, 1.357; *χ*^2^=29.342), indicating a statistically higher risk of diabetic retinopathy in the GLP1-ra and insulin cohort compared with the control cohort. The HR for DMO was 1.013 (95% CI 0.960, 1.069; *χ*^2^=20.611), indicating no significant difference between the two cohorts (Fig. 1, ESM Figs 2, 3).

#### GLP1-ra + insulin vs SGLT2i + insulin

The HR for diabetic retinopathy was 1.205 (95% CI 1.153, 1.259; *χ*^2^=1.818), indicating a higher risk of diabetic retinopathy in the GLP1-ra and insulin cohort compared with the SGLT2i and insulin cohort. For DMO, the HR was 1.130 (95% CI 1.056, 1.208; *χ*^2^=0.006), indicating a higher risk of DMO in the GLP1-ra and insulin cohort compared with the SGLT2i and insulin group (Fig. 1, ESM Figs 2, 3).

### Other outcomes: HF, hospitalisation and all-cause mortality

We conducted analyses of HF, hospitalisation (from any cause) and all-cause mortality as the direction of effect should particularly favour SGLT2i, given previously published RCT data [9–11]. The number of participants included in each analysis, number of individuals excluded due to having the outcome prior to the time window, number of events, median survival time and survival probability are displayed in ESM Tables 1–5.

#### SGLT2i + insulin vs control

The HR for HF was 0.805 (95% CI 0.784, 0.827; *χ*^2^=0.094), for hospitalisation was 0.711 (95% CI 0.703, 0.719; *χ*^2^=257.258) and for all-cause mortality was 0.543 (95% CI 0.532, 0.555; *χ*^2^=31.611), indicating lower risk of these outcomes in individuals on SGLT2i and insulin therapy.

#### GLP1-ra + insulin vs control

The HR for HF was 0.701 (95% CI 0.684, 0.719; *χ*^2^= 222.909), for hospitalisation was 0.558 (95% CI 0.552, 0.565; *χ*^2^=1469.193) and for all-cause mortality was 0.464 (95% CI 0.454, 0.474; *χ*^2^= 656.799), indicating lower risk of the outcomes in individuals on GLP1-ra and insulin therapy.

#### E-values

E-values for primary analyses are presented in ESM Table 6. In brief, E-values for SGLT2i + insulin vs control and GLP1-ra + insulin vs control analyses were generally greater than 1.5 (except for the diabetic retinopathy outcome in the SGLT2i + insulin vs control analysis which was 1.36). That is, an unmeasured confounder would need to have minimum strength of association with both the exposure and the outcome of greater than 1.5 to fully explain away the treatment–outcome association; we believe such a confounder is unlikely. However, unmeasured confounding may have influenced the result for the diabetic retinopathy outcome in the SGLT2i + insulin vs control analysis given the low E-value.

## Discussion

Diabetic retinopathy and DMO are leading causes of blindness worldwide. In this study, we explore the impact of SGLT2i and GLP1-ra therapy on the risk for developing diabetic retinopathy and DMO, through a real-world study from a global federated database, including around two million people with type 2 diabetes receiving insulin treatment. Our principal findings are as follows: (1) SGLT2i reduced the incidence of DMO, while GLP1-ra did not significantly affect DMO incidence; (2) SGLT2i resulted in a minimal but statistically significant increase in the risk of diabetic retinopathy; however, it is unlikely to be clinically significant and it should be interpreted in the context of multiple testing; GLP1-ra increased the incidence for diabetic retinopathy compared with a propensity score matched control cohort; and (3) direct comparison of SGLT2i and GLP1-ra therapies demonstrated that the GLP1-ra cohort had an increased risk for the development of both diabetic retinopathy and DMO. In keeping with well-established literature, SGLT2i and GLP1-ra were associated with a reduced risk of HF, hospitalisation and all-cause mortality [8, 9]. To the best of our knowledge, this is the largest real-world study to evaluate the benefits of SGLT2i and GLP1-ra on diabetic retinopathy and DMO in individuals with type 2 diabetes receiving insulin.

Although this is a retrospective study and thus randomisation and controlling of the confounding variables was not possible, we propensity score matched for a significant number of the key known confounding factors, which were well balanced amongst the comparison groups. Despite propensity score matching, unmeasured confounding variables may bias our findings. However, E-values of greater than 1.5 (for SGLT2i vs control and GLP1-ra vs control analyses) suggest that confounders with such magnitude of association with both exposure and outcome are unlikely. Propensity score matching could not account for duration/severity of diabetes in this platform, leading to the use of insulin treatment as an active comparator design to reduce the extent of confounding by indication. In our propensity score matching for HbA_1c_, BMI and eGFR, we opted for categorical instead of continuous values, as imputation for missing values was not computationally possible. Employing continuous values in complete case analyses would result in a reduction of sample size and statistical power. However, categorising data comes with limitation of residual confounding. This being a real-world federated database study, raw data were inaccessible due to confidentiality agreements. We obtained access to summary-level data relying on clinical ICD-10 coding, introducing some limitations. Due to lack of individual data, multivariant analysis is not possible. Data completeness cannot be assured, and the availability of 5 year data for all participants from the index event remains uncertain. Nonetheless, censoring was used to account for participant attrition from the cohort during the analysis, ensuring their exclusion from the statistical analysis. The diagnostic modality and severity of the main and positive control outcomes could not be established due to these constraints on data availability. The sequence of initiating insulin relative to GLP1-ra or SGLT2i is not known. Moreover, the length of treatment with SGLT2i, GLP1-ra and/or insulin or adherence to treatment and therefore the time of exposure to those medications are not known. All participants were taking insulin, a medication usually used in more advanced type 2 diabetes, and the results may not be generalisable to other patient populations. The dataset's majority being from the USA, with its insurance-based healthcare system, may affect generalisability when considering early insulin use due to cost considerations.

In a meta-analysis of RCTs, SGLT2i demonstrated a protective effect against retinal disease when directly compared with glucose-lowering agents [22]. A further systematic review and network meta-analysis suggested that canagliflozin increased diabetic retinopathy, although this signal was not present for two other SGLT2is (empagliflozin and dapagliflozin) [23]. Lin et al demonstrated the rate of developing diabetic retinopathy was comparable between individuals receiving SGLT2i and GLP1-ra, although individuals receiving SGLT2i had a lower risk of proliferative diabetic retinopathy [14]. Similar to our study, a post hoc analysis of the EMPA-REG OUTCOME trial showed no increased risk of retinopathy with empagliflozin vs placebo [15]. Other retrospective studies have shown favourable outcomes in individuals taking SGLT2i, either by slowing the progression of diabetic retinopathy or by reducing the risk for developing diabetic retinopathy, or both [15, 24]. In a multi-institutional cohort study, for example, Su et al observed a 25% reduced risk of developing DMO in individuals receiving SGLT2i compared with individuals receiving GLP1-ra [25]. Also, Tatsumi et al demonstrated that SGLT2i can reduce the risk of DMO (measured through central retinal thickness) in individuals with diabetic retinopathy [26].

Studies investigating the effect of GLP1-ra on diabetic ocular disease have also been heterogenous in nature. The SUSTAIN-6 RCT demonstrated that the rates of retinopathy complications, including vitreous haemorrhage, blindness or photocoagulation, were higher with semaglutide compared with placebo [27], although this trial was a CV outcome trial rather than a diabetic retinopathy-orientated trial. A systematic review and meta-analysis also suggested that subcutaneous semaglutide increased the risk of diabetic retinopathy, but this finding was not demonstrated with oral semaglutide [23]. The LEADER trial found a non-significant increased incidence of retinopathy in the GLP1-ra (liraglutide) group compared with placebo [28]. However, in nationwide cohort and Mendelian randomisation studies, both showed lower risk of diabetic retinopathy for individuals treated with GLP1-ra [29]. The AngioSafe Type 2 Diabetes Study demonstrated no association between GLP1-ra and severe diabetic retinopathy, as diagnosed with fundus imaging [30]. This has been further supported by a systematic review and meta-analysis which showed no increase in diabetic retinopathy risk in the GLP1-ra groups [31].

Diabetic retinopathy is strongly associated with other microangiopathies [7]. Understanding the effects of SGLT2i and GLP1-ra on diabetic retinopathy and DMO may delineate phenotypes and thus allow for risk stratification for the development of strategies to tackle wider diabetic (macro- and microvascular) complications. Gibbons and Freeman previously demonstrated that treatment-induced diabetic neuropathy (insulin neuritis) occurred in parallel with diabetic retinopathy and microalbuminuria, suggesting a common underlying pathophysiological mechanism [32]. Our data are consistent with several other studies showing a beneficial effect of SGLT2i and GLP1-ra on HF, hospitalisation and all-cause mortality. The EMPA-REG OUTCOME trial demonstrated that the empagliflozin cohort had a reduced risk for hospitalisation for HF and all-cause mortality compared with the placebo group [33]. Similarly, the DAPA-HF trial showed that dapagliflozin can prevent progression of disease in individuals with pre-existing HF, and reduce the risk of HF hospitalisations and CV death [34]. Conversely, the LEADER trial (GLP1-ra: liraglutide), PIONEER 6 trial (GLP1-ra: oral semaglutide) and EXSCEL trial (GLP1-ra: exenatide) described a significant reduction in all-cause mortality and a non-statistically significant reduction in hospitalisation for HF in the GLP1-ra group compared with placebo [28, 35, 36]. We recently showed that combination therapy (with SGLT2i and GLP1-ra) was associated with the greatest risk reduction in all-cause mortality vs a propensity score matched control cohort [19].

The mechanism through which SGLT2i translates to improved diabetic eye disease outcomes is likely multifactorial, although studies have suggested that it extends beyond glycaemic control. Hyperglycaemia results in oxidative stress, inflammation, vascular damage, capillary ischaemia and retinal tissue hypoxia [37]. SGLT2 transporters are present in retinal cells, including mesangial cells and retinal pericytes [38], which may suggest that SGLT2i agents act in directly reducing glucose levels in the retinal microcirculation and hence reduce glucotoxicity, oxidative stress and inflammation, and restore insulin signalling, halting glucose-induced vascular and endothelial dysfunction, progression of microangiopathy and, importantly, diabetic retinopathy [7, 39]. Moreover, SGLT2is have been demonstrated to reduce the total circulating volume, improve sodium balance and increase haematocrit, which may improve oxygen delivery to tissues [11, 40]. These effects may also influence risk of DMO. Regarding the GLP1-ra pharmacodynamics, a hypothesised mechanism through which GLP1-ra predisposes to diabetic retinopathy is via rapid reduction in HbA_1c_ which can lead to alterations in VEGF and IGF-1 that can lead to the development or early worsening of diabetic retinopathy [41, 42]. A Phase III clinical trial aiming to evaluate the effect of semaglutide on diabetic ocular disease is underway, with the results projected for 2027 [43].

The results of this study support the hypothesis that SGLT2i and GLP1-ra may have a beneficial role in preventing the development of DMO in individuals with type 2 diabetes taking insulin, but due to the study design they do not establish a causal link. There is a clear need to evaluate the impact of SGLT2i and GLP1-ra on diabetic eye disease in dedicated prospective studies, primarily to determine their ability to prevent and even treat DMO. Moreover, it is not known whether the association of GLP1-ra and diabetic retinopathy may be mitigated over time, as short-term tightening of diabetic control can precipitate worsening of diabetic retinopathy, but over the longer-term improved glycaemic control is beneficial in holistic diabetes outcomes.

## Conclusions

Our study demonstrates that SGLT2i therapy is associated with a reduced risk of DMO in individuals with type 2 diabetes taking insulin. However, SGLT2i use was not associated with an altered risk of diabetic retinopathy, while GLP1-ra use was associated with an increased risk for diabetic retinopathy. Direct comparison of SGLT2i and GLP1-ra suggests favourable outcomes with SGLT2i in both the development of DMO and diabetic retinopathy. Dedicated RCTs appear justified to determine whether SGLT2i can reduce the risk of potentially blinding diabetic eye disease, particularly in people who are at high risk of DMO.

### Supplementary Information

Below is the link to the electronic supplementary material.

Supplementary file1 (PDF 539 KB)

## Data Availability

To gain access to the data in the TriNetX research network, a request can be made to TriNetX (https://live.trinetx.com), but costs may be incurred, a data sharing agreement would be necessary and no patient identifiable information can be obtained.

## Abbreviations

CV: Cardiovascular
DMO: Diabetic macular oedema
EMR: Electronic medical record
GLP1-ra: Glucagon-like peptide 1 receptor agonist
HCO: Healthcare organisation
HF: Heart failure
ICD-10-CM: ICD, Tenth Revision, Clinical Modification
SGLT2: Sodium–glucose cotransporter 2
SGLT2i: Sodium–glucose cotransporter 2 inhibitor
VEGF: Vascular endothelial growth factor
