# Supplementary material for: Risk of diabetic retinopathy and diabetic macular oedema with sodium–glucose cotransporter 2 inhibitors and glucagon-like peptide 1 receptor agonists in type 2 diabetes: a real-world data study from a global federated database
Source: Diabetologia. 2024 Apr 8;67(7):1271–82. doi: 10.1007/s00125-024-06132-5 (PMC11153282; doi:10.1007/s00125-024-06132-5)

Electronic Supplementary Material

Supplementary Material Table 1: Diabetic retinopathy/ DMO outcomes in the SGLT2i + insulin cohort vs control

	SGLT2i	Control
**Diabetic Retinopathy**		
Number of patients	147,543	148,130
Number of events	3,262	4,305
Survival probability (%)	93.64	94.44
Number of patients excluded	6,995	6,408
**Macular oedema**		
Number of patients	150,688	150,612
Number of events	1,376	2,323
Survival probability (%)	97.35	97.05
Number of patients excluded	3,850	3,926

Supplementary Material Table 2: Diabetic Retinopathy/ DMO outcomes in the GLP1-ra + insulin cohort vs control

	GLP1-ra	Control
**Diabetic Retinopathy**		
Number of patients	173,723	174,727
Number of events	6,119	5,374
Survival probability (%)	92.37	94.39
Number of patients excluded	9,368	8,364
**Macular Oedema**		
Number of patients	178,062	178,022
Number of events	2,554	2,855
Survival probability (%)	96.87	97.09
Number of patients excluded	5,029	5,069

Supplementary Material Table 3: Diabetic Retinopathy/ DMO outcomes in the GLP1-ra + insulin vs SGLT2i + insulin cohorts.

	GLP1-ra	SGLT2i
**Diabetic Retinopathy**		
Number of patients	130,378	130,388
Number of events	4,981	3,307
Survival probability (%)	91.57	93.08
Number of patients excluded	8,739	8,729
**Macular Oedema**		
Number of patients	134,747	134,624
Number of events	2,055	1,449
Survival probability (%)	96.65	97.05
Number of patients excluded	4,370	4,493

Supplementary Material Table 4: Heart failure/Hospitalisation/All-cause Mortality outcomes in the SGLT2i + insulin cohort vs control

	SGLT2i	Control
**Heart Failure**		
Number of patients	107,794	120,950
Number of events	8,518	14,718
Survival probability (%)	83.06	79.43
Number of patients excluded	46,744	33,588
**Hospitalisation**		
Number of patients	154,538	154,538
Number of events	47,864	70,189
Survival probability (%)	48.77	39.51
**All-cause mortality**		
Number of patients	152,815	152,057
Number of events	12,151	30,152
Survival probability (%)	81.20	69.12
Number of patients excluded	1,723	2,481

Supplementary Material Table 5: Heart failure/Hospitalisation/All-cause Mortality outcomes in the GLP1-ra + insulin cohort vs control

	GLP1-ra	Control
**Heart Failure**		
Number of patients	163,143	156,539
Number of events	10,100	15,356
Survival probability (%)	87.14	83.74
Number of patients excluded	19,948	26,552
**Hospitalisation**		
Number of patients	183,091	183,091
Number of events	46,789	76,859
Survival probability (%)	58.60	44.06
**All-cause mortality**		
Number of patients	181,231	180,746
Number of events	11,005	26,775
Survival probability (%)	86.65	76.64
Number of patients excluded	1,860	2,345

Supplementary Material Table 6: E-values for the primary outcomes

	Diabetic Retinopathy	DMO
	E-value (CI)	E-value (CI)
SGLT2i vs control	1.36 (1.19)	1.68 (1.49)
GLP1-ra vs control	1.94 (1.83)	n/a
GLP1-ra vs SGLT2i	1.7 (1.57)	1.51 (1.3)

Supplementary Material Figure 1: Propensity Score Matching graphs

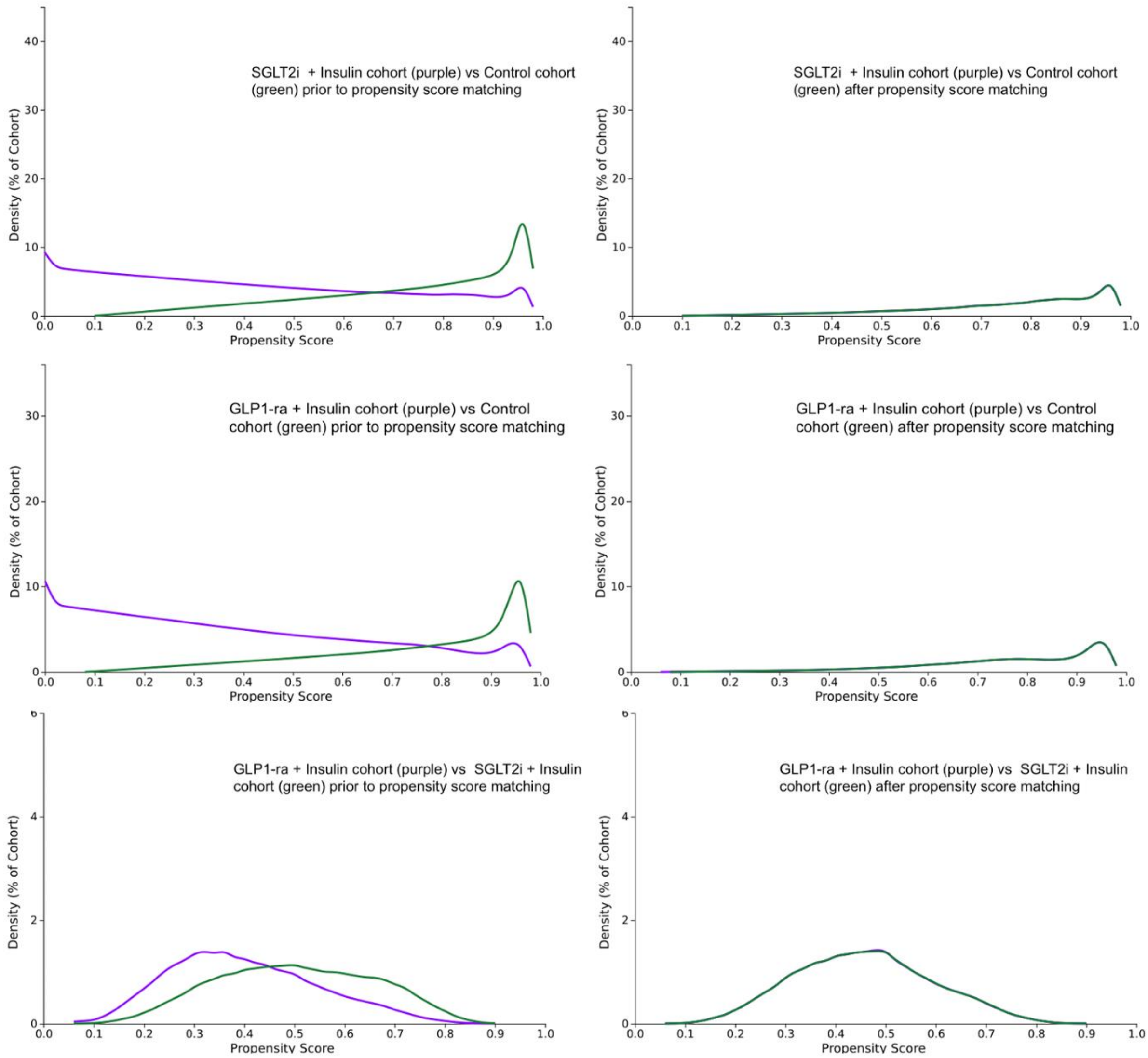

Supplementary Material Figure 2: Kaplan-Meier plots for DMO

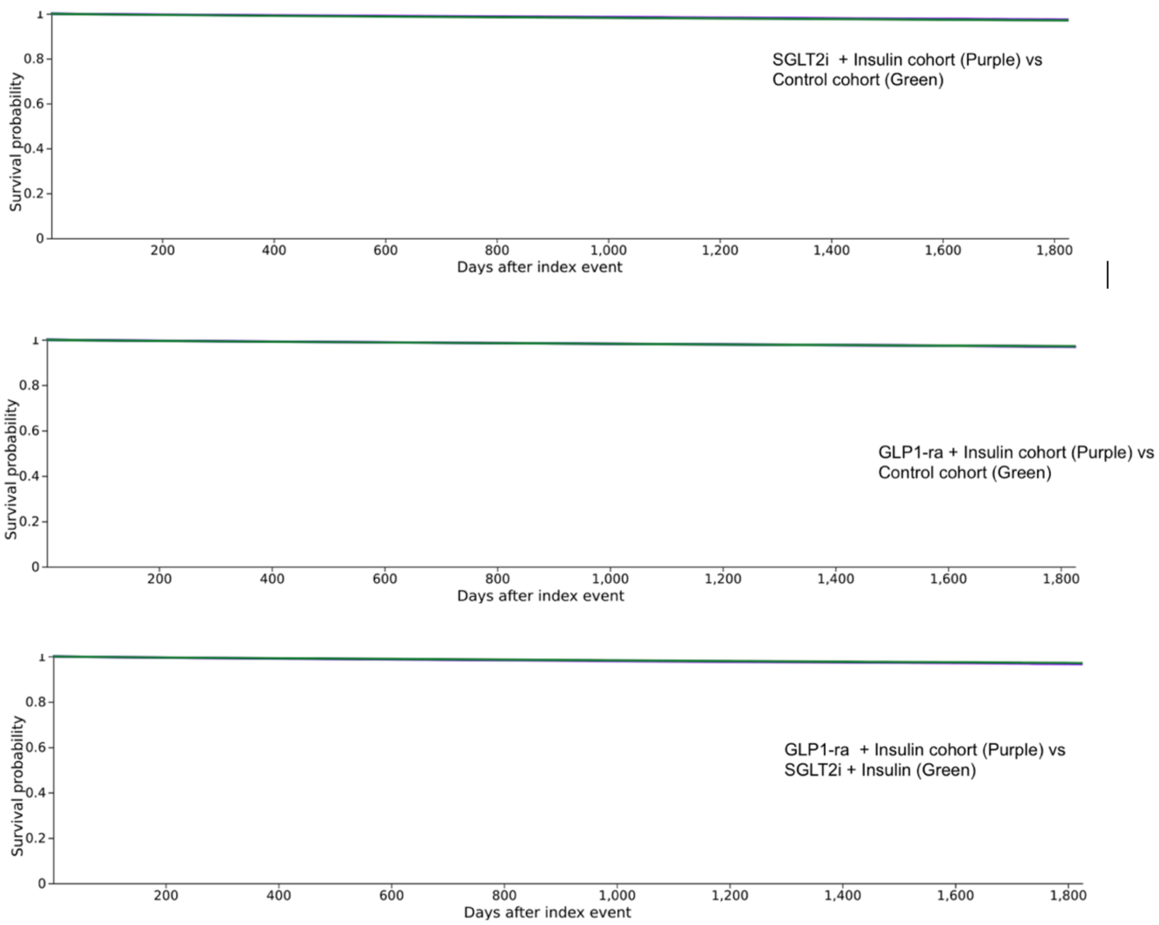

Supplementary Material Figure 3: Kaplan-Meier plots for Diabetic retinopathy

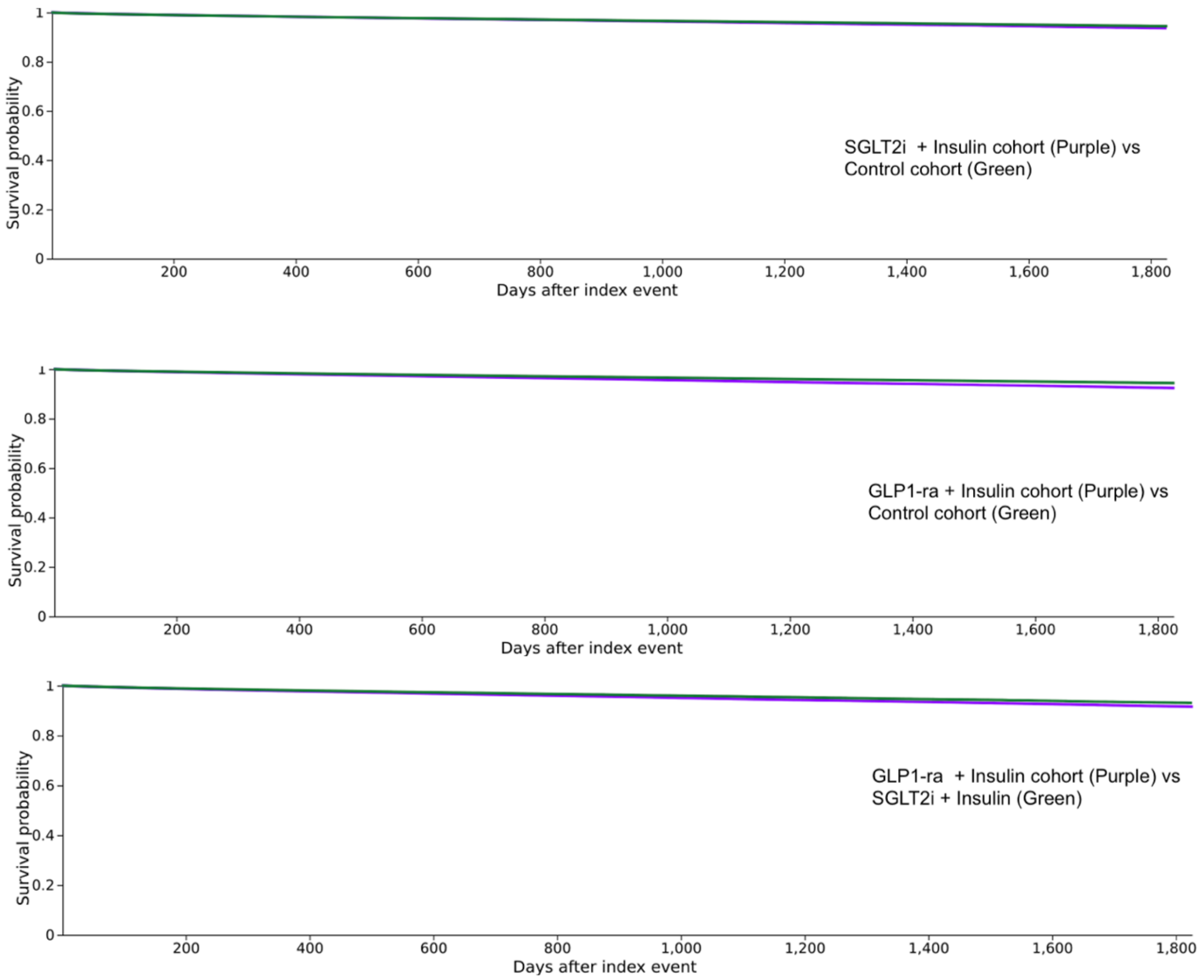

Supplement: Supplementary file 1 — Supplementary file1 (PDF 539 KB) [file 125_2024_6132_MOESM1_ESM.pdf]
